# Association of HIV status and treatment characteristics with VIA screening outcomes in Malawi: A retrospective analysis

**DOI:** 10.1371/journal.pone.0262904

**Published:** 2022-01-25

**Authors:** Samuel Lewis, Misheck Mphande, Florence Chibwana, Temwa Gumbo, Ben Allan Banda, Hitler Sigauke, Agnes Moses, Sundeep Gupta, Risa M. Hoffman, Corrina Moucheraud

**Affiliations:** 1 University of California Los Angeles David Geffen School of Medicine, Los Angeles, CA, United States of America; 2 Partners in Hope Medical Center, Lilongwe, Malawi; 3 Department of Health Policy and Management, University of California Los Angeles Fielding School of Public Health, Los Angeles, CA, United States of America; Baylor College of Medicine, UNITED STATES

## Abstract

**Background:**

Although evidence from high-resource settings indicates that women with HIV are at higher risk of acquiring high-risk HPV and developing cervical cancer, data from cervical cancer “screen and treat” programs using visual inspection with acetic acid (VIA) in lower-income countries have found mixed evidence about the association between HIV status and screening outcomes. Moreover, there is limited evidence regarding the effect of HIV-related characteristics (e.g., viral suppression, treatment factors) on screening outcomes in these high HIV burden settings.

**Methods:**

This study aimed to evaluate the relationship between HIV status, HIV treatment, and viral suppression with cervical cancer screening outcomes. Data from a “screen and treat” program based at a large, free antiretroviral therapy (ART) clinic in Lilongwe, Malawi was retrospectively analyzed to determine rates of abnormal VIA results and suspected cancer, and coverage of same-day treatment. Multivariate logistic regression assessed associations between screening outcomes and HIV status, and among women living with HIV, viremia, ART treatment duration and BMI.

**Results:**

Of 1405 women receiving first-time VIA screening between 2017–2019, 13 (0.9%) had suspected cancer and 68 (4.8%) had pre-cancerous lesions, of whom 50 (73.5%) received same-day lesion treatment. There was no significant association found between HIV status and screening outcomes. Among HIV+ women, abnormal VIA was positively associated with viral load ≥ 1000 copies/mL (aOR 3.02, 95% CI: 1.22, 7.49) and negatively associated with ART treatment duration (aOR 0.88 per additional year, 95% CI: 0.80, 0.98).

**Conclusion:**

In this population of women living with HIV with high rates of ART coverage and viral suppression, HIV status was not significantly associated with abnormal cervical cancer screening results. We hypothesize that ART treatment and viral suppression may mitigate the elevated risk of cervical cancer for women living with HIV, and we encourage further study on this relationship in high HIV burden settings.

## Introduction

Malawi has the greatest cervical cancer burden worldwide [1]. Screening is an effective approach to preventing the development of cervical cancer, and associated morbidity and mortality [2–4]. Malawi’s national guidelines recommend routine cervical cancer screening using visual inspection with acetic acid (VIA) for all women starting at age 25, once every 2 years for women living with HIV and every 3 years for women without HIV [5]. VIA is a validated, inexpensive, and feasible method of cervical cancer screening widely used in low resource settings, found to have both high sensitivity (60–87%) and specificity (64–95%) for detecting high grade cervical dysplasia and cancer [6–10]. In Malawi, women with VIA lesions suggestive of pre-cancer are treated using ablative methods, if eligible (cryotherapy or thermal ablation) (i.e., “screen and treat”), and women ineligible for ablation are referred for Loop Electrosurgical Excision Procedure (LEEP), or advanced care if cancer is suspected.

Between 2011–2015, the Malawi national program screened approximately 145,000 women using VIA, of whom 5.1% were found to have pre-cancerous lesions and 4.3% had suspected cancer [11]. Other studies from Malawi have similarly estimated an abnormal VIA rate (suspected pre-cancerous and cancerous lesions) between 3–6% in this period [12, 13]. This is lower than the pooled abnormal VIA estimate from a meta-analysis of studies from sub-Saharan Africa conducted from 1996–2014 (16.5%) [14]–although there is substantial variation in the region, ranging from 7% in Tanzania [15, 16] and 8% in Mozambique [17], to almost 20% in Zambia [18] and Kenya [19].

Although there is clinical evidence that women with HIV are at greater risk for the acquisition and progression of high-risk HPV types including development of pre-cancer and cancer [20, 21], evidence for an association between abnormal VIA and HIV status from “screen and treat” programs in sub-Saharan Africa is mixed. The Malawi national program found that women living with HIV had higher rates of pre-cancerous lesions (8.8% versus 5.0% in women without HIV, in 2015) and suspect cancer (6.4% versus 3.0%) [11]. Similarly, studies from Zambia [18, 22], Cameroon [23], and Tanzania [15, 24] have found that women living with HIV are more likely to have abnormal VIA than women without HIV, including in modeled estimates that adjusted for other important covariates–although the magnitude of the increased likelihood varied substantially across studies. In contrast, data from Nigeria [25] and Mozambique [17] did not find a significant association between HIV status and VIA outcome.

Additionally, there is mixed information about how specific sociodemographic and clinical characteristics of women with HIV influence cervical cancer screening outcomes [18, 22, 23, 26]. One study from South Africa found that advanced age, but not CD4 count, was associated with a higher rate of invasive cervical cancer among women living with HIV [27]; but another found that CD4 count was associated with abnormal cervical smear results [28].

We analyzed clinical data from a “screen and treat” program in Malawi. We had two research questions: (1) What were the program outcomes, i.e., abnormal VIA rate and uptake of treatment (lesion treatment); and how did these outcomes differ by HIV status? (2) What HIV-related characteristics were associated with abnormal VIA in this population?

## Materials and methods

### Study setting

Data were obtained from Partners in Hope Medical Center, which operates a large, free antiretroviral therapy (ART) clinic in Lilongwe, Malawi that also offers cervical cancer screening (VIA) and lesion treatment for eligible women (thermal ablation). Lesions were eligible for same day treatment if they were restricted to the cervix, involved less than 75% of the cervix, and were not suspicious for invasive cancer. Women with lesions which were not eligible for same day treatment were referred for further evaluation. These “screen and treat services” are offered free of charge to all women with HIV, and for a fee of K4800 (~$6.50) to women without HIV. VIA and thermal ablation were performed by experienced nurses with training in screening and treatment of cervical dysplasia.

### Data collection

Data were obtained from visits occurring between June 8, 2017 through November 28, 2019. Records were accessed by the research team between November 1 and December 23, 2019. Patient demographics (age, marital status, area of residence, HIV status) and screening information (reason for visit, screening result, management) were extracted from the clinic’s screening registry. The following data were collected: date of HIV diagnosis, body mass index (BMI), viral load (VL), and ART start date. For variables that may change over time (BMI, VL, and ART regimen), data were collected from the closest ART visit within 6 months of a woman’s cervical cancer screening date to reflect values proximate to the time of screening.

### Data analysis

For this analysis we focused on women receiving VIA for the first time. Screening results and management were described by HIV status. Screening results were categorized as suspected cancer, pre-cancerous lesions, normal VIA, and other (includes cervicitis, vaginal bleeding, polyps, and inability to visualize cervix). The association between HIV status and abnormal VIA screening (pre-cancerous lesion or suspected cancer) was assessed through logistic regression with adjustment for age and year of screening. Multivariate logistic regression was used to assess the associations between abnormal VIA and viral suppression (VL <1000 copies/mL), ART treatment duration, and BMI. Analyses were restricted to women with a VL and BMI measurement within 6 months of VIA screening date, and adjusted for age, year of screening, and duration on ART. CD4 count is not routinely collected in Malawi and was excluded from analysis. All analyses were conducted using Stata v16 software (StataCorp 2019).

### Ethical review and consent

The study protocol was approved by the Institutional Review Board at the University of California, Los Angeles, and the National Health Sciences Research Committee in Malawi. A consent waiver was obtained as all data was retrospectively collected and anonymized for analysis.

## Results

**Fig 1:**
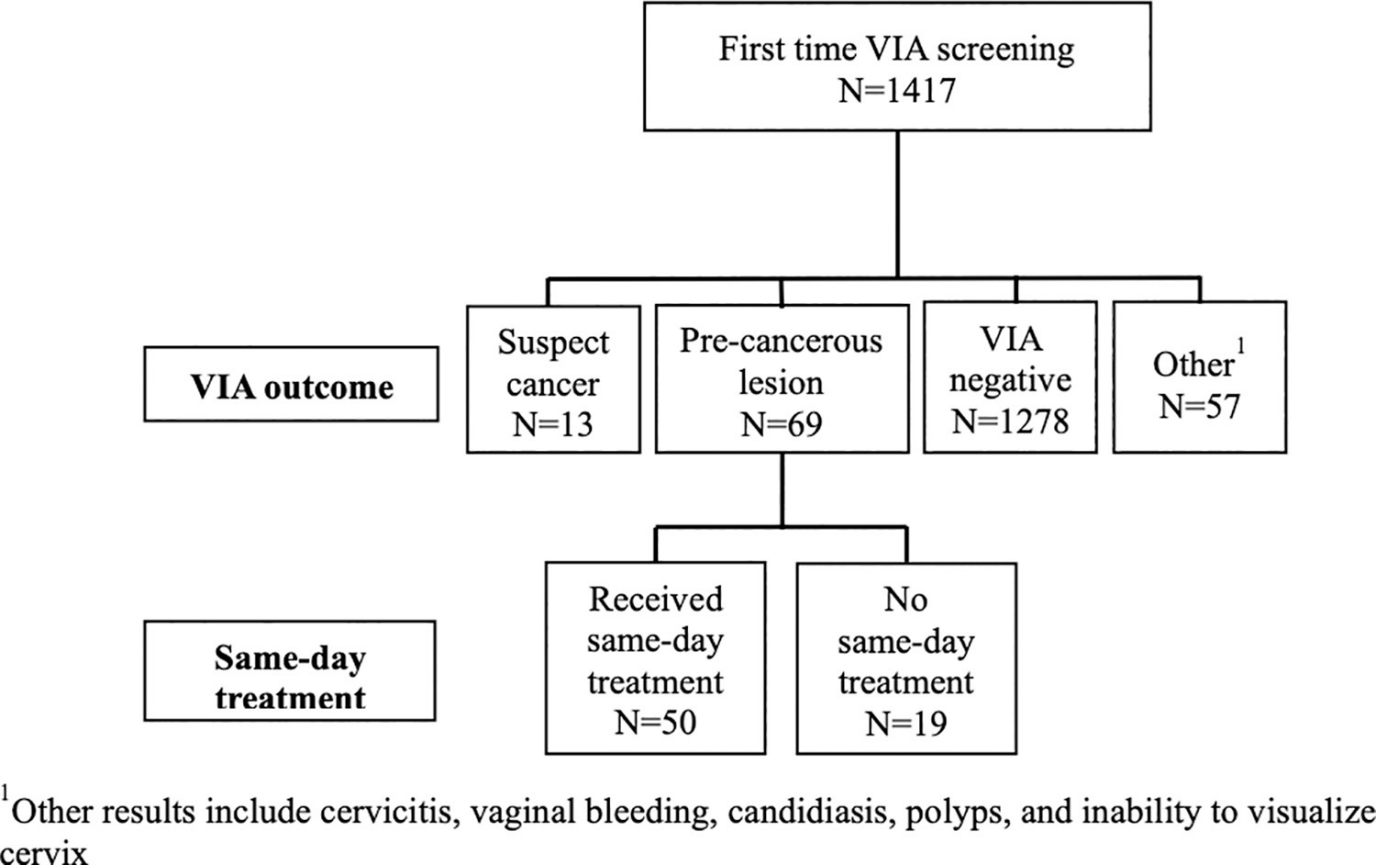
VIA screening visits, June 2017-October 2019: Results and same-day treatment flow chart.

Between June 2017 and October 2019, 1417 women received VIA for the first time, of which 1405 had known HIV status (Fig 1). Most of these women were aged 25–49 years (73.6%), and approximately half were married (Table 1). The proportion of study participants who were living with HIV increased over time, from 73.8% in 2017 to 96.1% in 2019. ART coverage among women living with HIV averaged 99.8% for the entire study period (data not shown).

**Table 1:** Characteristics of women undergoing first-time VIA, total and by HIV status.

	Total (n = 1405)	Women living with HIV (n = 1305)	Women without HIV (n = 100)
**Age, n (%)**			
< 25 years	60 (4.3%)	53 (4.1%)	7 (7.1%)
25–49 years	1033 (73.6%)	963 (73.8%)	70 (70.7%)
>49 years	311 (22.2%)	289 (22.2%)	22 (22.2%)
**Marital status, n (%)**			
Single	143 (10.2%)	120 (9.2%)	23 (23%)
Married	796 (56.7%)	727 (55.7%)	69 (69%)
Separated/divorced	212 (15.1%)	210 (16.1%)	2 (2.0%)
Widowed	254 (18.1%)	248 (19.0%)	6 (6.0%)
**Year of first VIA screening n (%)**			
2017	124 (8.8%)	96 (7.4%)	28 (28.0%)
2018	353 (25.1)	315 (24.1%)	38 (38.0%)
2019	928 (66.1%)	894 (68.5%)	34 (34.0%)

VIA, visual inspection with acetic acid; ART, antiretroviral therapy.

Overall, 5.8% of women screened for the first time had an abnormal screening result (either pre-cancerous lesions or suspected cancer). Among women living with HIV who were screened for the first time, 5.0% had pre-cancerous lesions and 1.0% had suspected cancer (Table 2). Among women without HIV, 3.0% had pre-cancerous lesions and there were no cases of suspected cancer. Most (72.3%) women living with HIV with pre-cancerous lesions received same-day treatment, as did 66.7% of women without HIV. Among all women with pre-cancerous lesions who did not receive same-day treatment, 55% were referred to higher care, 30% deferred for later treatment, and 15% were unspecified.

**Table 2:** VIA outcomes of women screened for the first time and association with HIV status (n = 1405).

	VIA outcomes	
	Women living with HIV	Women without HIV
**Screening result**	n = 1305	n = 100
Normal VIA	1176 (90.1%)	93 (93.0%)
Abnormal VIA	78 (6.0%)	3 (3.0%)
Pre-cancerous lesion, n (%)	65 (5.0%)	3 (3.0%)
Suspected cancer, n (%)	13 (1.0%)	0
Other^a^, n (%)	51 (3.9%)	4 (4.0%)
**Thermal ablation uptake among women with pre-cancerous lesions**	n = 65	n = 3
Received same-day thermal ablation	47 (72.3%)	2 (66.7%)
Postponed treatment	6 (9.2%)	0
Referred or missing^b^	12 (18.5%)	1 (33.3%)

VIA, visual inspection with acetic acid.

^a^ Other results include cervicitis, vaginal bleeding, candidiasis, polyps, and inability to visualize cervix.

^b^ Women with suspected cancer were not eligible for same-day treatment.

Pre-cancerous lesions were not significantly more common among women living with HIV than women without HIV in this sample, in unadjusted (OR 1.70, 95% CI: 0.52, 5.49) or adjusted (aOR 1.35, 95% CI: 0.41, 4.51) models (S1 Table). There was also no significant association between HIV status and the combined outcome of pre-cancerous lesion or suspected cancer (aOR 1.86, 95% CI: 0.57, 6.14).

Among women living with HIV in this sample with viral load data available, 92% (611/665) were virally suppressed (<1000 copies/mL) at the ART visit most proximal to VIA screening. The median time gap between VIA screening and viral load measure was 7.7 weeks (IQR: 2.9–16.7 weeks). Lack of viral suppression was positively and significantly associated with abnormal VIA, with 13% of women without viral suppression having abnormal VIA versus only 5% of women with viral suppression (aOR 3.02, 95% CI 1.22, 7.49) (Table 3). There was no apparent association between abnormal VIA and body mass index (aOR 1.47 for BMI 25.0–29.9, 95% CI 0.70,3.12). Longer duration on ART was negatively associated with abnormal VIA, with an adjusted OR of 0.88 for each additional year on treatment (95% CI 0.80, 0.98).

**Table 3:** Association between abnormal VIA (pre-cancerous or cancerous lesion) and HIV clinical characteristics among women living with HIV screened for the first time (n = 665).

	Abnormal VIA	Odds of abnormal VIA
	n (%)	aOR (95% CI; p-value)
Viral load (most recent within 6 months of VIA)^a^		
<1000 copies/mL	29 (4.8%)	Ref
≥1000 copies/mL	7 (13.0%)	3.02 (1.22, 7.49; p = 0.017)
Body mass index (most recent within 6 months of VIA)^a^		
<18.5	1 (2.1%)	0.40 (0.05, 3.12; p = 0.383)
18.5–24.9	17 (5.4%)	Ref
25.0–29.9	14 (7.2%)	1.47 (0.70, 3.12; p = 0.310)
≥30.0	4 (3.7%)	0.77 (0.25, 2.39; p = 0.652)
ART treatment duration^b^		
<5 years	16 (8.0%)	1.94 (0.97, 3.89; p = 0.062)
5 years	20 (4.3%)	Ref
Continuous		0.88 (0.80, 0.98; p = 0.024)

aOR, adjusted odds ratio; VIA, visual inspection with acetic acid; ART, antiretroviral therapy.

^a^Adjusted odds ratio controlling for age (categorical), year of screening, and duration on ART.

^b^Adjusted odds ratio controlling for age (categorical) and year of screening.

## Discussion

This study illustrates the outstanding potential of “screen and treat” in a low-resource setting: over 70% of VIA-positive women received same-day lesion treatment, which is nearly double the treatment completion rates that have been reported elsewhere including in Malawi and other countries in Africa, where rates have generally been reported to be less than 50% (range 31–61%) [11, 12, 17, 23, 29]. These high rates of uptake and completion of “screen and treat” may be because the program operates at an urban center of excellence in Malawi and receives financial support from PEPFAR/USAID for equipment, supplies, training, and nursing staff. Future studies might seek to assess what health system and service characteristics contribute to success of these programs to inform best practices for other facilities. Additionally, this program provided thermal ablation as the mode of treatment following abnormal VIA whereas the existing literature has focused on outcomes (including treatment uptake) in programs that use cryotherapy [30, 31]. Thermal ablation is a well-tolerated therapy and is less complex to deliver in a low-resource setting when compared to cryotherapy [17, 32–35]. More information is needed about outcomes from programs using thermal ablation, particularly with the WHO’s endorsement of thermal ablation as an acceptable alternative treatment approach in low-resource settings [36].

Although we found greater completion of same-day treatment for eligible women than in other studies, it was still not universal, and further work is needed to understand how to reach universal coverage of screen and treat among eligible women. Moreover, there was insufficient data to assess both adherence to follow-up care guidelines (i.e., VIA screening within 1 year of lesion treatment) and treatment efficacy. Other papers have documented deviations from clinical guidelines for cervical cancer screening including incorrect follow-up periods [37]. Further research is needed to evaluate reasons for low rates of follow-up and to develop and rigorously evaluate implementation strategies to improve adherence to evidence-based guidelines for “screen and treat” [38].

In this study, in which women living with HIV had high rates of ART coverage (99.8%) and viral suppression (92% with VL<1000 copies/mL), the rate of VIA positivity was slightly but insignificantly higher among women living with HIV compared to women without HIV (5.0% versus 3.0%). The existing literature contains mixed evidence about the association between VIA positivity and HIV status. Studies from Malawi [11], Zambia [18], Cameroon [22], and Tanzania [15, 24] have found a significant and positive association between being HIV positive and VIA positivity, with reported adjusted odds ratios ranging from 1.9 to 4.1. In contrast, large studies from Mozambique [17] and Nigeria [25] found no significant association between HIV status and cervical cancer screening outcomes. These variable findings by setting may relate to differences in clinical characteristics across populations of women living with HIV, such as duration on ART, CD4 counts, and viral suppression levels. Most existing studies, including those reporting both a positive association and no association between HIV status and VIA positivity, have not reported data on the study population’s level of HIV disease control [11, 15, 18, 22, 24]. This study’s findings support ART treatment and viral suppression as protective factors that may mitigate the elevated risk of cervical cancer among women living with HIV. Mixed evidence to date may thus reflect differences in these factors across countries and study period, especially as ART access expanded over time and as viral suppression increased accordingly [39].

There is strong evidence that ART use is associated with reduced prevalence of high-risk HPV and incidence of advanced cervical lesions, with greater protective effects associated with longer duration on ART [26]. However, fewer studies have explored the role of viral suppression. Our study found that viremia ≥1000 copies/mL was associated with abnormal VIA with an odds ratio of 3.5. Though our study did not have data on CD4 count because it is not routinely monitored in Malawi, previous studies have demonstrated an association between low CD4 count and abnormal VIA and cervical cancer [28, 40, 41]. In order to develop a more complete understanding of this relationship, future research on VIA “screen and treat” programs should include information about women’s clinical characteristics so we can better understand modifiable risk factors and target these to reduce risk for women living with HIV [42–45].

Some limitations to this analysis should be noted. First, this is a single-site study from a well-established, PEPFAR/USAID funded HIV program. Future analyses should incorporate data from diverse facilities in order to increase sample size and enhance generalizability. Second, the available information on women participating in “screen and treat” was relatively limited including lack of CD4 count, high rates of missing viral load data, and use of a viral suppression cut-off of 1,000 copies/mL (the Malawi guideline at the time under study). Of note, though the recommended frequency of routine viral load monitoring in Malawi at the time under study was every two years for individuals established on ART, we used a stricter inclusion criterion of viral load measurement within 6 months of screening, in order to have a more accurate assessment of viral suppression at the time of screening. We would encourage more in-depth data collection efforts including more information about sociodemographic characteristics in order to improve our understanding of disparities in screening and treatment, and about clinical characteristics for women with and without HIV. Third, many more women living with HIV were included than women without HIV. This is largely attributable to the setting of the study (a well-established ART clinic) and the availability of free screening for women living with HIV (versus equivalent of approximately $6.50 USD for those without HIV). Direct comparisons between these two populations were therefore limited, and may be biased if these populations were different in important ways e.g. due to this free structure. Future studies assessing outcomes based on HIV status should seek to identify more balanced and comparable populations. Finally, some women may have been misclassified as first time VIA screeners due to misreporting. There is a need for better longitudinal follow-up of women receiving VIA in order to reduce misclassifications as well as produce more accurate estimates of treatment completion and appropriate follow-up care.

## Conclusions

In this study, VIA “screen and treat” outcomes among a population predominantly composed of women living with HIV in Malawi were comparable to other large-scale VIA programs in the region. Uptake and completion of same-day treatment was substantially higher, although treatment efficacy and post-treatment follow-up care were unknown. High viral load, but not HIV status, was significantly associated with abnormal VIA. Further research is needed to better understand how duration on ART, virologic suppression, and other HIV-related clinical characteristics affect rates of abnormal VIA and treatment outcomes. As calls to eliminate global cervical cancer inequities grow, a more complete understanding of the interplay between HIV and cervical cancer is needed to guide efforts in nations facing high burdens of both diseases.

## Supporting information

S1 Table Association between HIV status and VIA outcomes among women screened for the first time (n = 1404). (TIF)
