# Supplementary figures and images for: Association of HIV status and treatment characteristics with VIA screening outcomes in Malawi: A retrospective analysis
Source: PLoS One. 2022 Jan 25;17(1):e0262904. doi: 10.1371/journal.pone.0262904 (PMC8789172; doi:10.1371/journal.pone.0262904)

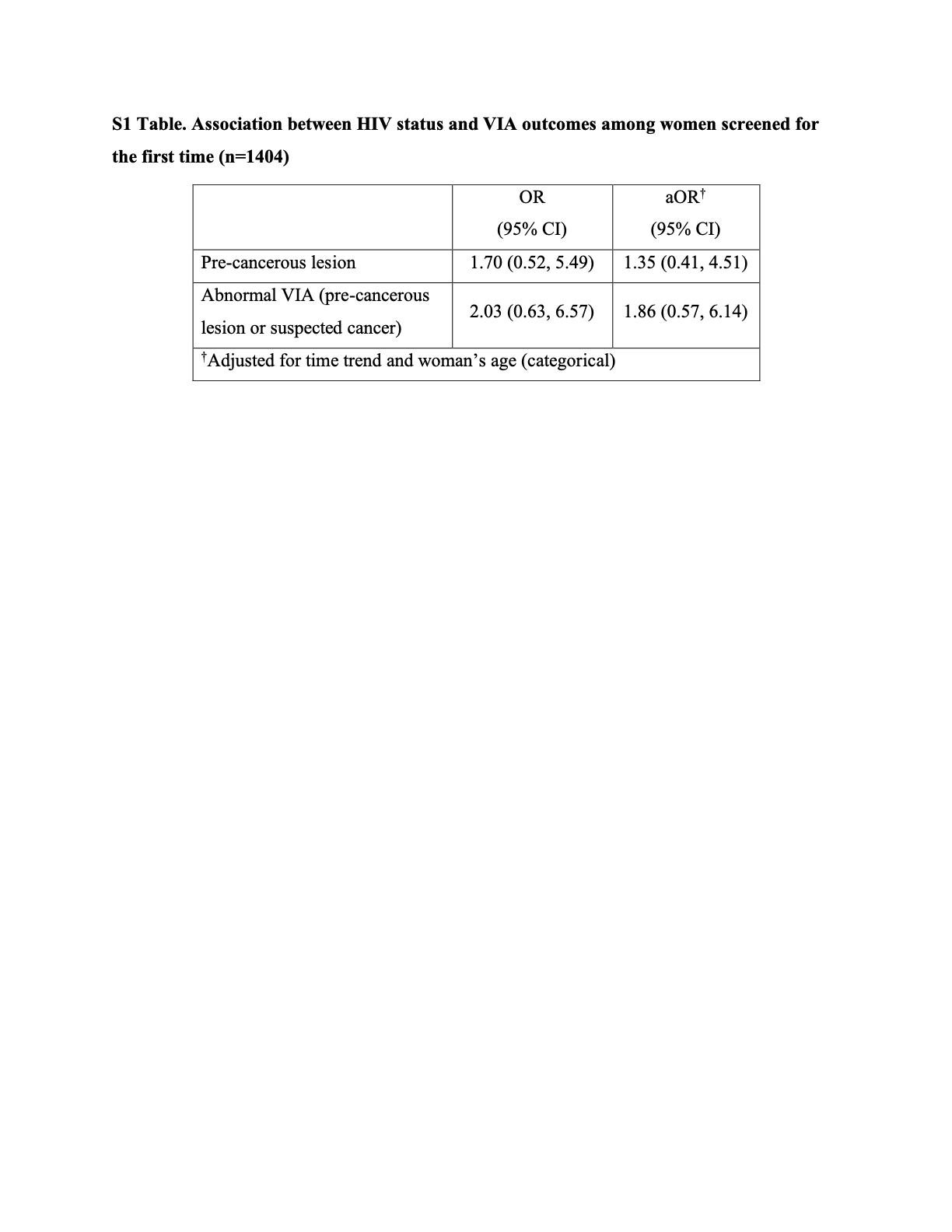

Supplement: S1 Table — (TIF) [file pone.0262904.s001.tif]
